# 2-Phenyl-*N*′-(2-phenyl­acet­yl)acetohydrazide

**DOI:** 10.1107/S1600536812019861

**Published:** 2012-05-12

**Authors:** Hatem A. Abdel-Aziz, Ching Kheng Quah, Hoong-Kun Fun

**Affiliations:** a Department of Pharmaceutical Chemistry, College of Pharmacy, King Saud University, PO Box 2457, Riyadh 11451, Saudi Arabia; b X-ray Crystallography Unit, School of Physics, Universiti Sains Malaysia, 11800 USM, Penang, Malaysia

## Abstract

In the title compound, C_16_H_16_N_2_O_2_, the *N*′-acetyl­acetohydrazide group is approximately planar (r.m.s. deviation = 0.018 Å for the eight non-H atoms) and makes dihedral angles of 81.92 (6) and 65.19 (6)° with the terminal phenyl rings. The phenyl rings form a dihedral angle of 62.60 (7)°. In the crystal, mol­ecules are linked into sheets lying parallel to (001) by N—H⋯O and C—H⋯O hydrogen bonds. One O atom accepts one N—H⋯O and one C—H⋯O hydrogen bond and the other O atom accepts one N—H⋯O and two C—H⋯O hydrogen bonds. The N—H⋯O hydrogen bonds lead to *R*_2_^2^(8) loops and the C—H⋯O hydrogen bonds generate *R*_2_^1^(6) loops.

## Related literature

For general background to and the pharmaceutical applications of hydrazine derivatives, see: Bredihhin & Mäeorg (2008 ▶); Ragnarsson (2001 ▶); Ling *et al.* (2001 ▶). For further synthesis details, see: Magedov & Smushkevich (1991 ▶). For standard bond-length data, see: Allen *et al.* (1987 ▶). For the stability of the temperature controller used in the data collection, see Cosier & Glazer (1986 ▶). For hydrogen-bond motifs, see: Bernstein *et al.* (1995 ▶).
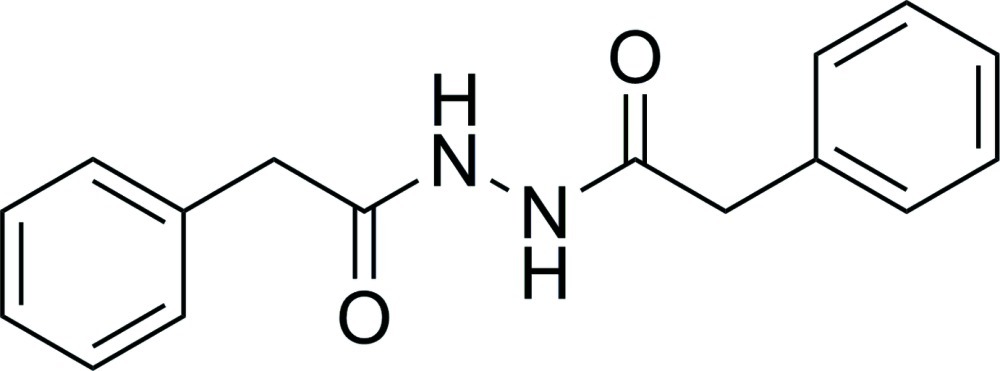


## Experimental

### 

#### Crystal data

C_16_H_16_N_2_O_2_
*M* *_r_* = 268.31
Triclinic, 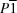

*a* = 5.4531 (6) Å
*b* = 7.9283 (9) Å
*c* = 15.1758 (17) Å
α = 94.271 (2)°
β = 92.613 (2)°
γ = 90.830 (2)°
*V* = 653.50 (13) Å^3^
*Z* = 2
Mo *K*α radiation
μ = 0.09 mm^−1^
*T* = 100 K
0.35 × 0.14 × 0.05 mm

#### Data collection

Bruker SMART APEXII DUO CCD diffractometer
Absorption correction: multi-scan (*SADABS*; Bruker, 2009 ▶) *T*_min_ = 0.968, *T*_max_ = 0.996
12991 measured reflections
3458 independent reflections
2428 reflections with *I* > 2σ(*I*)
*R*_int_ = 0.030

#### Refinement

*R*[*F* ^2^ > 2σ(*F* ^2^)] = 0.041
*wR*(*F* ^2^) = 0.134
*S* = 1.04
3458 reflections
245 parameters
All H-atom parameters refined
Δρ_max_ = 0.38 e Å^−3^
Δρ_min_ = −0.29 e Å^−3^

### 

Data collection: *APEX2* (Bruker, 2009 ▶); cell refinement: *SAINT* (Bruker, 2009 ▶); data reduction: *SAINT*; program(s) used to solve structure: *SHELXTL* (Sheldrick, 2008 ▶); program(s) used to refine structure: *SHELXTL*; molecular graphics: *SHELXTL*; software used to prepare material for publication: *SHELXTL* and *PLATON* (Spek, 2009 ▶).

## Supplementary Material

Crystal structure: contains datablock(s) global, I. DOI: 10.1107/S1600536812019861/hb6773sup1.cif

Structure factors: contains datablock(s) I. DOI: 10.1107/S1600536812019861/hb6773Isup2.hkl

Supplementary material file. DOI: 10.1107/S1600536812019861/hb6773Isup3.cml

Additional supplementary materials:  crystallographic information; 3D view; checkCIF report

## Figures and Tables

**Table 1:** Hydrogen-bond geometry (Å, °)

*D*—H⋯*A*	*D*—H	H⋯*A*	*D*⋯*A*	*D*—H⋯*A*
N2—H1*N*2⋯O1^i^	0.867 (16)	1.970 (16)	2.8076 (15)	162.4 (16)
C10—H10*A*⋯O1^i^	0.971 (16)	2.465 (16)	3.3103 (18)	145.3 (13)
N1—H1*N*1⋯O2^ii^	0.907 (16)	1.948 (16)	2.8283 (15)	163.2 (15)
C7—H7*A*⋯O2^ii^	0.973 (16)	2.479 (16)	3.3209 (18)	144.8 (13)
C7—H7*B*⋯O2^iii^	1.00 (2)	2.56 (2)	3.4929 (19)	155.2 (13)

Symmetry codes: (i) 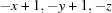; (ii) 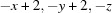; (iii) 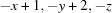.

**Fig. 1.**
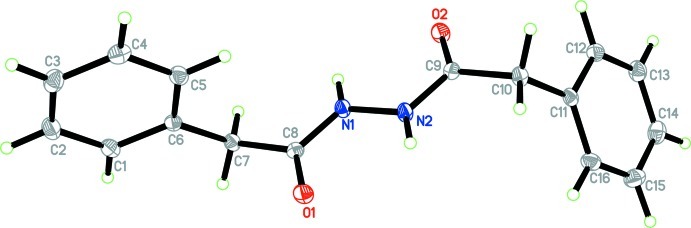
The molecular structure of the title compound showing 50% probability displacement ellipsoids for non-H atoms.

**Fig. 2.**
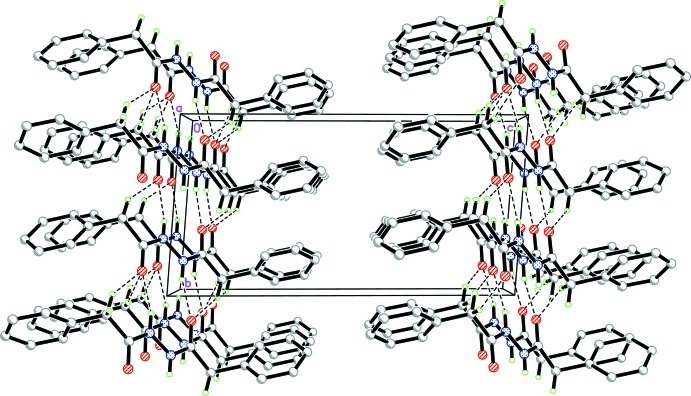
The crystal structure of the title compound, viewed along the a axis. H atoms not involved in hydrogen bonds (dashed lines) have been omitted for clarity.

## supplementary crystallographic information

### Comment

Hydrazine derivatives are widely used in the pharmaceutical applications and also as precursors in organic synthesis (Bredihhin & Mäeorg, 2008). Several hydrazine derivatives were shown to be effective for treatment of tuberculosis, Parkinson's disease and hypertension (Ragnarsson, 2001). Moreover, some hydrazines possess neuroprotective activity and are used as antidepressant drugs (Ling *et al.*, 2001).

In the title compound, Fig. 1, the *N*'-acetylacetohydrazide moiety (O1/O2/N1/N2/C7-C10) is approximately planar (r.m.s. deviation = 0.018 Å for the 8 non-H atoms) and makes dihedral angles of 81.92 (6) and 65.19 (6)° with the two terminal benzene rings (C1-C6 and C11-C16), respectively. The two benzene rings form a dihedral angle of 62.60 (7)°. Bond lengths (Allen *et al.*, 1987) and angles are within normal ranges.

In the crystal (Fig.2), molecules are linked into planes parallel to the (001) *via* intermolecular N1–H1N1···O2, C7–H7A···O2 and C7–H7B···O2 trifurcated acceptor bonds (Table 1) and N2–H1N2···O1 and C10–H10A···O1 bifurcated acceptor bonds (Table 1), generating R_2_^1^(6) ring motifs (Bernstein *et al.*, 1995).

### Experimental

The title compound was prepared by the reaction of 2-phenylacetyl chloride with 2-phenylacetohydrazide in the presence of sodium carbonate in water at 5-10 °C (Magedov & Smushkevich, 1991).

### Refinement

All H atoms were located in a difference Fourier map and refined freely with N–H = 0.869 (18)-0.908 (19) Å and C–H = 0.942 (19)-1.013 (18) Å.

### Crystal data

**Table:**

C_16_H_16_N_2_O_2_	*Z* = 2
*M**_r_* = 268.31	*F*(000) = 284
Triclinic, *P*1	*D*_x_ = 1.364 Mg m^−^^3^
Hall symbol: -P 1	Mo *K*α radiation, λ = 0.71073 Å
*a* = 5.4531 (6) Å	Cell parameters from 3410 reflections
*b* = 7.9283 (9) Å	θ = 4.0–30.0°
*c* = 15.1758 (17) Å	µ = 0.09 mm^−^^1^
α = 94.271 (2)°	*T* = 100 K
β = 92.613 (2)°	Needle, colourless
γ = 90.830 (2)°	0.35 × 0.14 × 0.05 mm
*V* = 653.50 (13) Å^3^	

### Data collection

**Table:**

Bruker SMART APEXII DUO CCD diffractometer	3458 independent reflections
Radiation source: fine-focus sealed tube	2428 reflections with *I* > 2σ(*I*)
Graphite monochromator	*R*_int_ = 0.030
φ and ω scans	θ_max_ = 29.0°, θ_min_ = 1.4°
Absorption correction: multi-scan (*SADABS*; Bruker, 2009)	*h* = −7→7
*T*_min_ = 0.968, *T*_max_ = 0.996	*k* = −10→10
12991 measured reflections	*l* = −20→20

### Refinement

**Table:**

Refinement on *F*^2^	Primary atom site location: structure-invariant direct methods
Least-squares matrix: full	Secondary atom site location: difference Fourier map
*R*[*F*^2^ > 2σ(*F*^2^)] = 0.041	Hydrogen site location: inferred from neighbouring sites
*wR*(*F*^2^) = 0.134	All H-atom parameters refined
*S* = 1.04	*w* = 1/[σ^2^(*F*_o_^2^) + (0.0709*P*)^2^ + 0.1901*P*] where *P* = (*F*_o_^2^ + 2*F*_c_^2^)/3
3458 reflections	(Δ/σ)_max_ = 0.001
245 parameters	Δρ_max_ = 0.38 e Å^−^^3^
0 restraints	Δρ_min_ = −0.29 e Å^−^^3^

### Special details

**Table:**

Experimental. The crystal was placed in the cold stream of an Oxford Cryosystems Cobra open-flow nitrogen cryostat (Cosier & Glazer, 1986) operating at 100.0 (1) K.
Geometry. All esds (except the esd in the dihedral angle between two l.s. planes) are estimated using the full covariance matrix. The cell esds are taken into account individually in the estimation of esds in distances, angles and torsion angles; correlations between esds in cell parameters are only used when they are defined by crystal symmetry. An approximate (isotropic) treatment of cell esds is used for estimating esds involving l.s. planes.
Refinement. Refinement of F^2^ against ALL reflections. The weighted R-factor wR and goodness of fit S are based on F^2^, conventional R-factors R are based on F, with F set to zero for negative F^2^. The threshold expression of F^2^ > 2sigma(F^2^) is used only for calculating R-factors(gt) etc. and is not relevant to the choice of reflections for refinement. R-factors based on F^2^ are statistically about twice as large as those based on F, and R- factors based on ALL data will be even larger.

### Fractional atomic coordinates and isotropic or equivalent isotropic displacement parameters (Å^2^)

**Table:**

	*x*	*y*	*z*	*U*_iso_*/*U*_eq_	
O1	0.40065 (18)	0.64320 (12)	0.06665 (6)	0.0165 (2)	
O2	1.08994 (18)	0.85895 (12)	−0.07188 (6)	0.0166 (2)	
N1	0.7067 (2)	0.81353 (14)	0.02864 (7)	0.0131 (2)	
N2	0.7869 (2)	0.68791 (14)	−0.03185 (7)	0.0136 (2)	
C1	0.2796 (3)	0.94134 (17)	0.29275 (9)	0.0154 (3)	
C2	0.3026 (3)	0.90738 (18)	0.38118 (9)	0.0185 (3)	
C3	0.4978 (3)	0.81340 (18)	0.41175 (9)	0.0184 (3)	
C4	0.6710 (3)	0.75379 (18)	0.35344 (9)	0.0180 (3)	
C5	0.6502 (2)	0.78955 (17)	0.26515 (9)	0.0153 (3)	
C6	0.4540 (2)	0.88282 (16)	0.23362 (8)	0.0127 (3)	
C7	0.4336 (3)	0.92581 (17)	0.13786 (8)	0.0129 (3)	
C8	0.5093 (2)	0.78171 (16)	0.07486 (8)	0.0120 (3)	
C9	0.9839 (2)	0.71983 (16)	−0.07846 (8)	0.0120 (3)	
C10	1.0630 (3)	0.57244 (17)	−0.13972 (8)	0.0137 (3)	
C11	1.0445 (2)	0.61346 (16)	−0.23584 (8)	0.0127 (3)	
C12	1.2278 (3)	0.70965 (18)	−0.27062 (9)	0.0162 (3)	
C13	1.2100 (3)	0.74792 (18)	−0.35846 (9)	0.0186 (3)	
C14	1.0109 (3)	0.68811 (19)	−0.41296 (9)	0.0200 (3)	
C15	0.8286 (3)	0.59203 (19)	−0.37894 (9)	0.0196 (3)	
C16	0.8446 (3)	0.55437 (17)	−0.29055 (9)	0.0161 (3)	
H1N1	0.787 (3)	0.915 (2)	0.0321 (11)	0.023 (5)*	
H1N2	0.705 (3)	0.593 (2)	−0.0350 (11)	0.017 (4)*	
H1A	0.148 (3)	1.007 (2)	0.2720 (11)	0.022 (4)*	
H2A	0.175 (3)	0.951 (2)	0.4219 (10)	0.017 (4)*	
H3A	0.519 (4)	0.786 (2)	0.4710 (13)	0.031 (5)*	
H4A	0.809 (3)	0.686 (2)	0.3727 (11)	0.021 (4)*	
H5A	0.775 (3)	0.747 (2)	0.2245 (11)	0.018 (4)*	
H7A	0.529 (3)	1.028 (2)	0.1304 (10)	0.018 (4)*	
H7B	0.259 (4)	0.953 (2)	0.1223 (12)	0.027 (5)*	
H10A	0.967 (3)	0.471 (2)	−0.1318 (10)	0.016 (4)*	
H10B	1.240 (3)	0.554 (2)	−0.1200 (11)	0.021 (4)*	
H12A	1.373 (3)	0.757 (2)	−0.2322 (11)	0.022 (4)*	
H13A	1.339 (3)	0.814 (2)	−0.3815 (12)	0.029 (5)*	
H14A	1.002 (3)	0.713 (2)	−0.4733 (12)	0.028 (5)*	
H16A	0.720 (3)	0.489 (2)	−0.2662 (12)	0.029 (5)*	
H15A	0.692 (3)	0.549 (2)	−0.4190 (11)	0.017 (4)*	

### Atomic displacement parameters (Å^2^)

**Table:**

	*U*^11^	*U*^22^	*U*^33^	*U*^12^	*U*^13^	*U*^23^
O1	0.0172 (5)	0.0123 (5)	0.0198 (5)	−0.0052 (4)	0.0047 (4)	−0.0010 (4)
O2	0.0172 (5)	0.0119 (5)	0.0206 (5)	−0.0033 (4)	0.0051 (4)	−0.0013 (4)
N1	0.0151 (6)	0.0101 (5)	0.0141 (5)	−0.0019 (4)	0.0042 (4)	−0.0016 (4)
N2	0.0156 (6)	0.0100 (5)	0.0150 (5)	−0.0023 (4)	0.0052 (4)	−0.0021 (4)
C1	0.0137 (6)	0.0143 (6)	0.0184 (6)	−0.0001 (5)	0.0029 (5)	0.0002 (5)
C2	0.0195 (7)	0.0175 (7)	0.0185 (7)	−0.0024 (5)	0.0059 (5)	−0.0006 (5)
C3	0.0235 (8)	0.0176 (7)	0.0143 (6)	−0.0031 (6)	0.0014 (5)	0.0021 (5)
C4	0.0170 (7)	0.0173 (7)	0.0191 (7)	0.0005 (5)	−0.0028 (5)	0.0005 (5)
C5	0.0142 (6)	0.0146 (6)	0.0169 (6)	0.0002 (5)	0.0022 (5)	−0.0011 (5)
C6	0.0128 (6)	0.0107 (6)	0.0143 (6)	−0.0038 (5)	0.0017 (5)	−0.0007 (5)
C7	0.0139 (6)	0.0106 (6)	0.0143 (6)	0.0000 (5)	0.0028 (5)	−0.0003 (5)
C8	0.0124 (6)	0.0114 (6)	0.0121 (6)	−0.0005 (5)	−0.0006 (5)	0.0013 (4)
C9	0.0134 (6)	0.0112 (6)	0.0115 (6)	−0.0001 (5)	0.0012 (5)	0.0013 (4)
C10	0.0156 (7)	0.0107 (6)	0.0149 (6)	−0.0003 (5)	0.0039 (5)	0.0007 (5)
C11	0.0132 (6)	0.0090 (6)	0.0161 (6)	0.0016 (5)	0.0037 (5)	−0.0008 (5)
C12	0.0148 (7)	0.0155 (6)	0.0179 (6)	−0.0020 (5)	0.0026 (5)	−0.0013 (5)
C13	0.0211 (7)	0.0169 (7)	0.0182 (7)	−0.0019 (5)	0.0060 (5)	0.0009 (5)
C14	0.0254 (8)	0.0191 (7)	0.0155 (6)	0.0040 (6)	0.0016 (6)	0.0016 (5)
C15	0.0185 (7)	0.0185 (7)	0.0210 (7)	0.0010 (6)	−0.0043 (6)	−0.0005 (5)
C16	0.0131 (6)	0.0140 (6)	0.0212 (7)	−0.0018 (5)	0.0020 (5)	0.0012 (5)

### Geometric parameters (Å, º)

**Table:**

O1—C8	1.2355 (16)	C7—C8	1.5089 (18)
O2—C9	1.2332 (16)	C7—H7A	0.975 (18)
N1—C8	1.3426 (16)	C7—H7B	1.002 (19)
N1—N2	1.3922 (14)	C9—C10	1.5183 (18)
N1—H1N1	0.908 (19)	C10—C11	1.5172 (18)
N2—C9	1.3443 (16)	C10—H10A	0.972 (17)
N2—H1N2	0.869 (18)	C10—H10B	1.013 (18)
C1—C2	1.3888 (19)	C11—C16	1.3934 (19)
C1—C6	1.3977 (18)	C11—C12	1.3951 (18)
C1—H1A	0.950 (18)	C12—C13	1.3884 (19)
C2—C3	1.390 (2)	C12—H12A	1.009 (18)
C2—H2A	1.000 (16)	C13—C14	1.390 (2)
C3—C4	1.388 (2)	C13—H13A	0.963 (19)
C3—H3A	0.942 (19)	C14—C15	1.385 (2)
C4—C5	1.3899 (19)	C14—H14A	0.949 (18)
C4—H4A	0.977 (17)	C15—C16	1.3948 (19)
C5—C6	1.3960 (18)	C15—H15A	0.980 (17)
C5—H5A	0.986 (17)	C16—H16A	0.954 (19)
C6—C7	1.5167 (18)		
			
C8—N1—N2	118.83 (11)	O1—C8—N1	121.88 (12)
C8—N1—H1N1	123.8 (11)	O1—C8—C7	122.94 (12)
N2—N1—H1N1	117.2 (11)	N1—C8—C7	115.18 (11)
C9—N2—N1	118.90 (11)	O2—C9—N2	122.08 (12)
C9—N2—H1N2	125.2 (11)	O2—C9—C10	123.08 (12)
N1—N2—H1N2	115.9 (11)	N2—C9—C10	114.84 (11)
C2—C1—C6	120.35 (13)	C11—C10—C9	111.62 (11)
C2—C1—H1A	120.4 (10)	C11—C10—H10A	110.1 (9)
C6—C1—H1A	119.2 (10)	C9—C10—H10A	111.1 (10)
C1—C2—C3	120.42 (13)	C11—C10—H10B	110.7 (10)
C1—C2—H2A	118.5 (9)	C9—C10—H10B	104.3 (10)
C3—C2—H2A	121.0 (9)	H10A—C10—H10B	108.8 (14)
C4—C3—C2	119.67 (13)	C16—C11—C12	119.24 (12)
C4—C3—H3A	117.1 (12)	C16—C11—C10	120.29 (12)
C2—C3—H3A	123.2 (12)	C12—C11—C10	120.47 (12)
C3—C4—C5	120.00 (13)	C13—C12—C11	120.42 (14)
C3—C4—H4A	121.6 (10)	C13—C12—H12A	118.5 (10)
C5—C4—H4A	118.4 (10)	C11—C12—H12A	121.0 (10)
C4—C5—C6	120.81 (12)	C12—C13—C14	120.18 (13)
C4—C5—H5A	119.2 (9)	C12—C13—H13A	119.3 (11)
C6—C5—H5A	120.0 (9)	C14—C13—H13A	120.5 (11)
C5—C6—C1	118.74 (12)	C15—C14—C13	119.70 (13)
C5—C6—C7	121.05 (11)	C15—C14—H14A	120.6 (12)
C1—C6—C7	120.17 (12)	C13—C14—H14A	119.7 (12)
C8—C7—C6	112.43 (11)	C14—C15—C16	120.35 (14)
C8—C7—H7A	111.1 (10)	C14—C15—H15A	118.1 (10)
C6—C7—H7A	110.0 (9)	C16—C15—H15A	121.5 (10)
C8—C7—H7B	108.1 (11)	C11—C16—C15	120.10 (13)
C6—C7—H7B	108.9 (10)	C11—C16—H16A	118.7 (11)
H7A—C7—H7B	105.9 (14)	C15—C16—H16A	121.2 (11)
			
C8—N1—N2—C9	179.72 (11)	N1—N2—C9—O2	−2.93 (19)
C6—C1—C2—C3	−0.6 (2)	N1—N2—C9—C10	177.53 (11)
C1—C2—C3—C4	0.2 (2)	O2—C9—C10—C11	−61.91 (17)
C2—C3—C4—C5	0.6 (2)	N2—C9—C10—C11	117.63 (13)
C3—C4—C5—C6	−1.1 (2)	C9—C10—C11—C16	−100.39 (15)
C4—C5—C6—C1	0.6 (2)	C9—C10—C11—C12	80.05 (15)
C4—C5—C6—C7	178.53 (13)	C16—C11—C12—C13	0.8 (2)
C2—C1—C6—C5	0.2 (2)	C10—C11—C12—C13	−179.64 (12)
C2—C1—C6—C7	−177.69 (13)	C11—C12—C13—C14	−1.1 (2)
C5—C6—C7—C8	39.28 (18)	C12—C13—C14—C15	0.9 (2)
C1—C6—C7—C8	−142.87 (13)	C13—C14—C15—C16	−0.3 (2)
N2—N1—C8—O1	1.69 (19)	C12—C11—C16—C15	−0.3 (2)
N2—N1—C8—C7	−179.17 (11)	C10—C11—C16—C15	−179.84 (12)
C6—C7—C8—O1	62.54 (17)	C14—C15—C16—C11	0.1 (2)
C6—C7—C8—N1	−116.59 (13)		

### Hydrogen-bond geometry (Å, º)

**Table:**

*D*—H···*A*	*D*—H	H···*A*	*D*···*A*	*D*—H···*A*
N2—H1*N*2···O1^i^	0.867 (16)	1.970 (16)	2.8076 (15)	162.4 (16)
C10—H10*A*···O1^i^	0.971 (16)	2.465 (16)	3.3103 (18)	145.3 (13)
N1—H1*N*1···O2^ii^	0.907 (16)	1.948 (16)	2.8283 (15)	163.2 (15)
C7—H7*A*···O2^ii^	0.973 (16)	2.479 (16)	3.3209 (18)	144.8 (13)
C7—H7*B*···O2^iii^	1.00 (2)	2.56 (2)	3.4929 (19)	155.2 (13)

Symmetry codes: (i) −*x*+1, −*y*+1, −*z*; (ii) −*x*+2, −*y*+2, −*z*; (iii) −*x*+1, −*y*+2, −*z*.
